# Did Persian Nowruz Aggravate the 2019 Coronavirus Disease Crisis in Iran?

**DOI:** 10.1017/dmp.2020.178

**Published:** 2020-05-29

**Authors:** Mohammad Heidari, Nasrin Sayfouri

**Affiliations:** Community-Oriented Nursing Midwifery Research Center, Shahrekord University of Medical Sciences, Shahrekord, Iran; Department of Foreign Languages, School of Health Management and Information Sciences, Iran University of Medical Sciences, Tehran, Iran

**Keywords:** coronavirus, COVID-19, Iran, Persian Nowruz

Following the outbreak of the coronavirus disease (COVID-19) crisis in early 2020 in China, Iran was officially confirming some cases of infection on February 19, 2020, in Ghom – a religious city only 148 kilometers away from Tehran – as a result of the arrival of some flights from China to the city. Almost 2 weeks later, the Iranian Ministry of Health declared that severe acute respiratory syndrome coronavirus 2 (SARS-CoV-2) has been transmitted to most of the Iranian provinces.^1^

Due to the rapid epidemic nature of SARS-CoV-2, identified as the cause of COVID-19,^2^ most public places, including schools, higher education institutions, cinema and theater halls, concerts, and athletic leagues and matches, were closed and working hours of essential businesses were reduced. Disinfection of public places was started extensively by community-oriented organizations, and body temperature screening stations were launched at different entrances. To lessen social commuting and to break the transmission chain of COVID-19, self-quarantine orders, stay-at-home notices, and leaves of absence were issued and partially implemented in the country. The people were asked not to leave their homes unless they have urgent affairs. They were invited to cooperate with the authorities so that, together, they could fulfill the “We-Defeat-Corona” slogan.

With the above-mentioned measures, the degree of the outbreak was expected to be kept manageable. However, there was a matter about which all of the authorities in political, health, and treatment sectors were extremely concerned, that is, the imminent Iranian New Year (*Nowruz*) annual holiday and ceremonies on March 20, 2020, amid the outbreak of COVID-19. In Iranian culture, from the ancient times, it has been customary to visit relatives and friends or to take a trip during the Nowruz holidays. There was the threat that performing these ceremonies weaken the stay-at-home notice since visiting others and taking trips could aggressively speed up the spread of the disease. Using mass media and environmental posters, the authorities warned people not to take trips nor visit others and to postpone these ceremonies until post-corona era.

However, with the beginning of the Nowruz holidays, the police reported high traffic jams around the entrances of the Northern provinces of Iran.^3^ This was indicative of people’s inattentiveness to the warnings that ultimately resulted in the high incidence of COVID-19 in the Northern provinces (Gilan and Mazandaran).^1^ The former prediction, therefore, came true. On March 26, 2020, around the end of the Nowruz holidays, the trend of infection with SARS-CoV-2 in Iran showed a substantial increase, considerably beyond expectations (Figure 1).^4^ This indicates that the rate of infected individuals after the holidays has escalated daily while manifesting tangible differences compared with the rate of infected people before the ceremonies.

**FIGURE 1:**
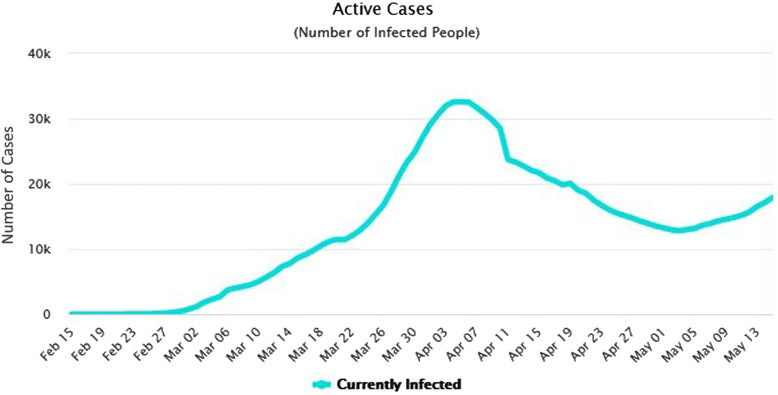
COVID-19 Infection Trend in Iran From February 15 to May 13, 2020.

As observed in Figure 1, the climax of the infection occurs in the first week of April, which is 2 weeks after the usual Nowruz trip-taking events, confirming the incubation period of the virus being between 3 and 14 days.^5^ Having considered this exponential trend, the social distancing order was issued on April 11 and was immediately implemented; citizens were told to observe the lockdown requirement, as well as the required distancing of at least 6 feet from others.

Another aspect of this order was that citizens were not permitted to exit the city of their residence nor to enter any other city hoping that the execution of this order can culminate in the reduction of COVID-19 cases in Iran.^6^ From the social distancing order date to the first days of May 2020, the morbidity and mortality of COVID-19 decreased constantly. Nevertheless, the sharp fall in the morbidity rate observed in Figure 1 during the first week of April could have been due to another reason, that is, people returning home from the Nowruz holidays resulting in a halt of the outbreak.

Nonetheless, in order to successfully resume economic activities, another order was issued on April 17, 2020, called *Smart Distancing Order*, according to which the low-risk occupations, groups, and gatherings were permitted to restart their activities, provided that they observe the required distancing.^7^ This permission has most likely been the cause of another rise of infections from the first week of May onward (Figure 1). This new rise has kept the educational centers at all levels still closed while involved teachers and students are continuing their online educational activities, which began during mid-February 2020.

To date, in Iran, the morbidity and mortality rates of COVID-19 have exceeded 120 000 and 7000, respectively.^1^ The trend of this prevalence is varied and, in some provinces, such as Khouzestan, the disease still shows high contagion. With regard to the unpredictability of the disease, the people in all regions of the country are warned to continue taking COVID-19 seriously and following the hygienic protocols strictly.

## References

[1] Ministry of Health & Medical Education (MOHME). Coronavirus epidemic in Iran. http://ird.behdasht.gov.ir/. Accessed May 17, 2020.

[2] World Health Organization. Coronavirus disease (COVID-19) pandemic. 2020 https://www.who.int/emergencies/diseases/novel-coronavirus-2019. Accessed May 17, 2020.

[3] Eghtesad Online News. Nowruz holidays. 2020 https://www.eghtesadonline.com. Accessed May 17, 2020.

[4] Worldometer. Coronavirus Iran. 2020. https://www.worldometers.info/coronavirus/country/iran/. Accessed May 17, 2020.

[5] He X , Lau EH , Wu P , et al. Temporal dynamics in viral shedding and transmissibility of COVID-19. Nat Med. 2020;26(5):672-675. 32296168 10.1038/s41591-020-0869-5

[6] Mashregh News. The start time of the social distancing plan has been announced. April 27, 2016. https://www.mashreghnews.ir/news/1055420. Accessed May 17, 2020.

[7] TRT. The beginning of smart distance in Iran. 2020. https://www.trt.net.tr/persian/yrn/2020/04/10/agz-fslh-gdhry-hwshmnd-dr-yrn-z-shnbh-23-frwrdyn-1399-1395428. Accessed May 17, 2020.

